# The Health and Environment Network and its achievements

**DOI:** 10.1186/1476-069X-11-S1-S1

**Published:** 2012-06-28

**Authors:** Alena Bartonova, Janna G Koppe, Aleksandra Fucic, Arno C Gutleb, Peter van den Hazel, Hans Keune

**Affiliations:** 1 NILU – Norwegian Institute for Air Research, Kjeller, Norway; 2 Institute for Medical Research and Occupational Health, Zagreb, Croatia; 3 Department of Environment and Agro-biotechnologies (EVA), Centre de Recherche Public - Gabriel Lippmann, Luxembourg; 4 Hulpverlening Gelderland Midden Public Health Services, The Netherlands; 5 Research Institute for Nature and Forest (INBO), Brussels; Centre of Expertise for Environment and Health, Faculty of Political and Social Sciences, University of Antwerp; naXys, Namur Center for Complex Systems, University of Namur, Belgium; 6 Ecobaby Foundation, Loenersloot, The Netherlands

## Why HENVINET

Environmental health is a rapidly developing field of great societal importance. New insights show that environmental influences, already in the early beginning of human life, determine health conditions later in life. The Health and Environment Network (HENVINET; 2006-2010) aimed to establish an integrated network to connect experts, policymakers and stakeholders addressing the environmental, social, political, climate and xenobiotic influences on human health.

The project started against the background of two foregoing EU initiatives that were addressing the problems of environment and health in children. The first was the European Strategy for Environment and Health, jointly prepared by four Directorate-Generals (DG Environment, DG RTD, DG Health and Consumers and the Joint Research Centre). The strategy was launched as the SCALE initiative (Science, Children, Awareness, Legislation and Evaluation) in 2003. Together with the following European Environment and Health Action Program 2004-2010, it aimed to bring the fields of environment and health closer to each other and develop recommendations for research and policy actions within four priority themes: childhood respiratory diseases, asthma and allergies, neurodevelopmental disorders, childhood cancer, and disruption of the endocrine system.

The second initiative PINCHE (2003-2005) (Policy Interpretation Network on Children’s Health and Environment) reviewed these four themes in relation to the aspects of exposure and health effects on children, discussing several compounds including dioxins, PCB’s, heavy metals (mercury, lead and cadmium), and pesticides, with the aim to formulate priorities in the political field.

In the HENVINET project, the four SCALE themes were taken up again, incorporating new insight on the importance of epigenetic changes, taking place already immediately after conception, caused by environmental factors and determining later functioning. This insight has changed our way of thinking, from the more thematic approach used in SCALE and PINCHE to the study of changes of DNA under the influence of endocrine and developmental disruptors. A separate new important issue, potential toxicity of nanomaterials and nanoparticles involving new and challenging pathways of toxicity, was included in the project at the mid-term.

## Why this supplement

This supplement presents 18 papers that cover the methods used and work done in the project. They describe the different ways how multi-disciplinary work was carried out in the different parts of the project, the results that were achieved, offer a theoretical view on decision-making complexity, and discuss our experience with social networking in a professional context. The largest space is devoted to case studies that expand expert elicitation through (1) the development of a causal diagram to visualise the links between environmental changes and their consequences on health, and (2) a format on how to carry out expert workshop on policy interpretation, resulting in policy briefs. Other work addresses further issues in complex decision-making: synthesis of research results in fields where decision-making will be required in a near future, translation of knowledge using decision support tools, and dialogue with decision makers where the starting point is a vision of health and environment target for the future. Space is also devoted to the description of how the consortium dealt with the inter-disciplinary and cross-sectoral communication issues, internally in the project, and in interactions with external experts and other stakeholders. The insights offered by social scientific reflection on how to deal with complexity in environmental health were essential to the progress of this work.

Inter-disciplinary approaches and the need to engage with societal and political issues are crucial parts of today’s research and scientific work. In this supplement, we want to share our experiences and results with professionals in medicine, epidemiology, toxicology and social sciences, and we hope also to awaken interest of students, paramedical people and others involved in addressing health and environment problems. Our work indicates that in the future, the priority of the Council of the EU in the field of environment and health should be oriented to the endocrine (i.e. developmental) disruptors and nanomaterials, with the aim of prevention or repair of genomic and non-genomic damage or other new effects. The way to address these issues requires collaboration of many disciplines. We have illustrated some of the difficulties in such inter-disciplinary work, and suggested how to overcome them, hoping that these experiences will be useful to the whole health and environment community.

## Competing interests

There are no competing interests.

